# Suspiciousness

**Published:** 1867-05

**Authors:** 


### SUSPICIOUSNESS.

One of the most laughable and amusing occupations of a publisher is reading letters from country folk in reference to orders for books, magazines, newspapers, &c. The extreme readiness to jump at the idea that somebody wants to cheat them out of the worth of two cents is amazing. They hear so much of the rascalities of large cities that they become afraid of their own shadow; being entirely oblivious of the fact that four times out of five the thousand and one little meannesses of which they read have been perpetrated by their own friends and neighbors and relations, who have come to New York to try their hands at all sorts of ill doings, but have'nt sense enough to keep themselves from being found out. If a New Yorker commits a lapse it is either a "big thing," or there is so much daring about it that more or less of admiration is felt for the fellow in the very few cases in which he is found out. Not long ago we knew of one whose best suit of clothes had gone to seed and who wanted to replenish. He came to New York with a young cart load of recommendations from dead editors and newspapers which had "gone up" twenty-five years before. He was patriotic; got hold of the ladies, and proposed an exhibition of his powers, the entire proceeds to be handed over to the Soldier's Relief Fund. Great preparations were made. Wives who had nothing better to do, taking young widows who could kill two birds with one stone, and bright-eyed girls who had "loveyers" in the wars, trudged around day after day selling tickets of admission, and things were going on swimmingly until about three hours before the performances were to have commenced, when the chief lady manager received a note saying that the operator had ordered a complete suit of broadcloth, and that the tailor had refused to deliver it until paid for, that he had been disappointed in some expected "remittances," that it would be impossible for him to appear unless he could have his new suit, and that if the bill could be paid for him he would refund at his earliest convenience. "Of course," said the dear, delightful, confiding creatures who composed the management, "we must pay the bill and he will pay it back; it shows that he has self respect, and that he feels it due to appear in becoming style." Well, the curtain rose, there was the suit of broadcloth, a perfect fit, and all glossy with its newness; inside the clothing was the man, and inside the man about a barrel of brandy, "be the same more or less," for there was no convenient opportunity for measuring the amount with precision; but if the volumn of screech, yell and roar was an index, an amount of liquor had been consumed by the person which would be perfectly appalling if it had been known; the curtain opportunely fell, and neither the suit nor what it covered were ever heard of more.

Some time ago, when we edited two publications, some one from down east wrote to know if a few specimen numbers could be sent him, that ours was a very reliable publication; that he had some influence in the neighborhood and could get a good lot of subscribers. In about two weeks the very same person, in the very same words, wrote for some specimen copies of the other monthly, not knowing that both came from the same office; and no doubt he thus obtained loads of publications from all parts of the country and thus kept himself posted as to literary matters at but little cost. It is a more common thing however for country people to read the notices of monthlies, and when they see that a subject is treated about which they would like to read they post off a letter in this wise: "I could easily get up a club for you, if I had a specimen number to show; the last one would perhaps be as good as any." It is needless to say that none of these writers are ever heard from again, unless under some other name or other post office address, to get another number. We venture to say that not over one person in ten who writes for a specimen number "with a view to subscribing," excepting those who honestly send the price [illegible] ever subscribes, in our experience.

But look at the other side of these country people, who as a class are the closest people in the world,—we mean the farmers of the United States. If a paper is offered to them at about half its price and about actual cost, they will think over and over again, and after great tribulation they bring forth the dirtiest dollar out of their filthy pocket books, always being particular to send the worst looking, the most torn and mutilated one they can find. They take it to the post master and in the presence of a dozen loafers in the "country store," where most post offices are kept, they state that money is enclosed; and with great misgivings deposit the valuable missive in its proper receptacle, and slowly wind their way home wondering all the time if they will ever hear of that precious dollar again; then they begin to calculate as closely as possible how long it will take to get to New York and how long to return, allowing ten minutes for delivery and answer; for they seem to suppose that somebody is at the office waiting for the letter, that there is no other letter but that, and all that the publisher has to do is to open, read and answer it without taking time to draw his breath. Having fixed to their own satisfaction the time when an answer should come, and knowing the day and hour that the only mail which ever comes to the village reaches them, they go two or three hours beforehand, hoping that the mail will come in that day sooner than usual, and they sit on some stump or the head of a whisky barrel, chew tobacco, spit and wait. Meanwhile they talk a little about the weather and the crops, but they soon run out of that, and knowing very little about any thing else they lapse again into silent chewing and spitting, once in a while stretching up and asking somebody what can be the reason the mail don't come. When it does come they await its opening with intense interest, being the only interest they have at that time, their whole souls being concentrated in the one fear that they have been caught in a trap; and when they see Tom, Dick and Harry of their neighbors, each carrying home a letter or opening an ample paper, they feel just as if they were nobody, as if not a soul on earth cared enough about them to even send them a printed circular, "price two cents;" then their little minds go on a raid in the half suspicion that they have been cheated, they work themselves up into the tallest kind of frenzy; by the time they get home they are so brimful of gall that they would burst if they did not vent it on somebody or something; if the tom cat happened to be in their way, or a puppy, they would give it such a kick as to send it yowling a mile beyond their cow-house; then down they sit to a scrawl which looks as if a cockroach had fallen into the inkstand and was making his escape across a piece of white paper; and now just hear some of their rants, literally. A stupid old dutchman writes to a cotemporary: "I sent you my money; you must have got it, pecause I put it mit my letter into the post office; so I know you must have got it; and if I don't get your paper right away I vill bublish you all over this land, so I vill." Another comes from a Connecticutcheon boy, the same place where wooden nutmegs come from, plaster of Paris hams and all that kind of honorable articles of commerce. The January, February and March numbers had been bound in one cover, as they treated pretty much on the same subjects, and would save postage to the subscribers besides making a valuable book of itself. "I inquired for my Journal of Health and was told that the January number was marked from January to March; and so one number of the Journal must answer for three months. How is this? Do not the proprietors of Hall's Journal of Health wish to do business in an honorable manner? I have not taken the Journal from the post office, nor do I intend to until it comes as it should. I can spare the money I sent you, if necessary. I had rather receive no compensation at all than a partial one. Trusting, however, that it is only an oversight and that you mean to do just as you agreed to, I am respectfully yours."

It may be well to state that the cause of delay in receiving the papers subscribed for in connection with the Journal of Health, was that by an inadvertance or misapprehension one list of names sent to that paper in due time to be copied on their books was returned to the office of the Journal of Health without belng copied, the mistake not having been discovered until several complaints had been sent in.

---

H. A. BUTTOLPH, M.D., Superintendent of the New Jersey Lunatic Asylum, has sent in his annual report to Governor

Ward at Trenton. It is a sad record; 367 patients in December, 1866; 540 had been under treatment during the year; 2,774 had been admitted since the institution was opened, May 15, 1848. The whole report is curious and instructive, and shows a scientific and practical ability highly creditable to the superintendent and his business management.

